# Complications Requiring Cochlear Reimplantation

**Published:** 2012

**Authors:** Seied Basir Hashemi, Hajar Bahrani fard

**Affiliations:** 1 *Department of Otolaryngology and Head and Neck Surgery, Khalili Hospital, Shiraz University of Medical Science, Shiraz, Iran*

**Keywords:** Cochlear implantation, Reimplantation, Revision surgery

## Abstract

**Introduction:**

Surgery for revision of cochlear implantation is an unusual but not uncommon occurrence. The purpose of this study was to discuss the major complications of cochlear implantation that we observed in 11out of 275 patients who underwent cochlear implantation in Khalili Hospital at Shiraz University of Medical Sciences.

**Materials and Methods:**

A retrospective analysis of 275patients who underwent cochlear implantation between 2003 and 2009 was performed. All of the patients were followed for one to five years.

**Results:**

Only 11 patients needed to undergo revision surgery or take medication. Out of those, five patients were referred with signs of device failure, all of whom underwent reimplantation. For the rest of the patients with complications, one patient underwent surgery due to a misplaced electrode, two patients had poor telemetry due to migration of the implant magnet, one patient presented with meningitis that was managed by medical treatment, and two patients presented with a massive scalp hematoma that responded to medical treatment.

**Conclusions:**

In our study the most common cause of a need for cochlear reimplantation was device failure.

## Introduction

Cochlear implantation has revolutionized the treatment of profound hearing loss. The procedure is relatively safe and has become the treatment of choice for both children and adults. Most patients who receive cochlear implantation show improved quality of life, speech recognition and communication. However, cochlear implantation has complications, which can be categorized as major or minor, depending on whether they require surgery, are life threatening or can be treated medically. Major complications include facial nerve palsy, meningitis, flap problems, device malfunction, infection of the middle ear cleft and device migration. Minor complications include transient facial palsy, small scalp hematoma and edema, facial stimulation, and pain that can be relieved by electrode deactivation. Both types of complications have economic burdens (1,2).

The purpose of this study was to discuss the major complications observed in 11 out of 275 (4%) patients who underwent cochlear implantation in Khalili Hospital at Shiraz University of Medical Sciences.

## Materials and Methods

A retrospective analysis of 275 patients who underwent cochlear implantation between 2003 and 2009 was performed. All patients were examined annually. The kinds of prostheses used in our center were the Combi 40 (Med EL) in 11 patients and in the others, a Nucleus implant (Cochlear Company). All the patients were followed for one to five years with a mean follow-up period of three years. Device failure that occurred due to abnormal implant performance or was detected by a failed integrity test despite correct electrode positioning was verified by radiography. Neuroresponse telemetry testing was also used to determine device failure in patients. Following detection of device failure, surgery and medical treatment were used for the observed major and minor complications.

## Results

This study included 275 patients who underwent surgery between 2003 and 2010 in the Ear, Nose and Throat Department of Khalili Hospital at Shiraz University of Medical Sciences. There were 135 male patients (49.1%) and 140 female patients (50.9%) with an age range of between 2 and 60 years old. Only 22 cases of hearing impairment that were treated were post lingual. All of the patients were visited annually with a mean follow-uptime of3 years (range: 1–5 years).

The complications observed in patients were categorized as major or minor. Major complications occurred in 11 patients (4.0%). Reaction to sutures at the site of implantation was the most minor complication and resulted in pain and in flammation in patients. This problem was improved with removal of the sutures and medical treatment. Sudden hearing lossdue to device failure was reported by 5 patients (1.81%), and after reimplantation all of them had a good response. Only 2patients (0.72%) had poor results from neuroresponse telemetry, indicating migration of the implant magnet. 

Meningitis occurred in 1patient (0.36%); a three-year-old boy who complained of headache, nausea and vomiting. Culture of his cerebrospinal fluids indicated the presence of streptococcus pneumonia. He was successfully treated with intravenous combined antibiotic therapy and over a follow-upperiod of 5 years had no further problems. One patient (0.36%) with a misplaced electrode underwent revision surgery to correct the electrode position (Medel) with a good response. Two patients (0.72%) develop a scalp hematoma, which was managed by repeated aspiration and application of a pressure dressing. 

## Discussion

Hearing aids are the principle means of auditory rehabilitation for patients with sensor neural hearing lost. Hearing aids are also the main mode of management of conductive hearing loss. Implantable hearing aids have several advantages for patients with hearing loss such as in creasedgain and dynamic range, reduced feedback, reduced maintenance, improved appearance and freedom from ear canal occlusion(3).Cochlear implantation is a safe procedure, where the rate of severe complications is below 2%.The success rate of cochlear implantation is due to a thorough consideration of the indications, limitations and risks. The complications that occur differ between children and adults (4,5).

The first cases of reimplantation were reported in 1985, with good results. In comparison with the literature, our rate of revision surgery is lower than has previously been reported. In an article in 2009, the overall rate of revision was reported to be 5.5%, with the rate in children being 7.3% and in adults 3.8% (6). Why the rate of revision surgery was significantly higher in children was not clear. Other studies reported the rate of revision surgery in children to be between 5.6% and 14%, and in adults to be between 5.4% and 7% (7-9). In our study the rate of revision surgery was 2.9% and all of the procedures occurred in children. Why the revision surgery occurred only in children is not clear, maybe this was due to the fact that most of our patients were children. 

In a large multi-institutional series on pediatric cochlear implantation, hard failure was the most common cause (46%) and in 41% of these cases a history of trauma was in evidence (10). In other studies hard failure was also the most common cause of reimplantation (8,10,11). In our study, the most common cause of reimplantation was soft failure (45% of all the cases that had complications and 1.8% of all the case sof implantation). In another study, the mean time of device failure was 7.6 years in children (12). However, in our study the minimum time for failure was 3 months and the maximum was 12 months after implantation. Meningitis is another major complication of cochlear implantationandis a critical situation. In other studies, the rate of meningitis post-operation was reported to be 0.3% to 0.5% (4,13). In our study the rate of this complication wasalso0.3%.

## Conclusion

Revision surgery for cochlear implantation is not uncommon, and in this regard consultation with parents regarding the possibility of device failure and other complications of the initial implantation is necessary.
